# Construction of a Mutant Library of *Avibacterium paragallinarum* Transposons and Screening and Preliminary Study of Genes Related to Biofilm Formation

**DOI:** 10.3390/microorganisms14040783

**Published:** 2026-03-30

**Authors:** Bingbing Fan, Qishuang Su, Yan Shao, Weidong Sun, Jingming Zhou, Xiangan Han, Wei Jiang

**Affiliations:** 1 Shanghai Veterinary Research Institute, Chinese Academy of Agricultural Sciences, Shanghai 200241, China; 13673763602@163.com (B.F.); 18584894886@163.com (Q.S.); shaoyan0220@163.com (Y.S.); 2 College of Veterinary Medicine, Nanjing Agricultural University, Nanjing 210095, China; swd100@njau.edu.cn; 3 Longhu Laboratory, Zhengzhou 450046, China; zhjingming@126.com

**Keywords:** *Avibacterium paragallinarum*, transposon, random mutant library, biofilm

## Abstract

*Avibacterium paragallinarum* (*Av. paragallinarum*), the causative agent of infectious coryza, imposes substantial economic burdens on the poultry industry by inducing growth retardation in broilers and reducing egg production in laying hens by up to 40%. Disease control is hindered by the limited efficacy of available vaccines and the increasing prevalence of antibiotic resistance—challenges that are exacerbated by the pathogen’s capacity to form biofilms, which facilitate bacterial persistence and enhance drug tolerance. To systematically elucidate the genetic determinants underlying biofilm formation in *Av. Paragallinarum*, we constructed a high-density random mutant library using mini-Tn5 transposon mutagenesis, comprising 3106 individual mutants. Phenotypic screening via crystal violet staining identified 188 mutants displaying altered biofilm-forming capacity relative to the wild-type strain, including 172 with enhanced and 16 with reduced biofilm formation. Sequencing of transposon insertion sites in these mutants revealed 105 disrupted genes involved in diverse biological pathways, including amino acid metabolism, quorum sensing, and transmembrane transport. A representative subset of eight mutants was selected for detailed phenotypic characterization. Their biofilm phenotypes were consistent with the initial screening results; certain mutants exhibited markedly enhanced biofilm formation (e.g., Tn-2206), whereas others, including Tn-1504, Tn-2428, and Tn-2859, showed significant reductions in biofilm production. Notably, these three biofilm-deficient mutants—harboring disruptions in a TonB-dependent receptor (Tn-1504), a GntP family permease (Tn-2428), and a hypothetical protein (Tn-2859)—displayed drastically attenuated virulence in vitro. Compared with the wild-type strain, these mutants exhibited reductions in cytotoxicity (up to 66.38%), cell adhesion (up to 50.68%), and invasive capacity, while maintaining normal growth kinetics. These findings indicate that the identified genes may play crucial roles in biofilm-associated virulence and highlight Tn-1504, Tn-2428, and Tn-2859 as promising candidates for the development of live attenuated vaccines. Collectively, this study provides a comprehensive genetic foundation for the rational design of novel anti-biofilm strategies against *Av. paragallinarum*.

## 1. Introduction

*Av. paragallinarum*, a Gram-negative bacterium, is the causative agent of infectious coryza (IC), a prevalent respiratory disease in chickens that leads to substantial economic losses in the poultry industry worldwide. The disease was first described in 1920, and the etiological agent was subsequently isolated in 1932. IC is clinically characterized by facial swelling, nasal discharge, and respiratory distress, which collectively result in growth retardation in broilers and a marked reduction (10–40%) in egg production in laying hens [1,2,3,4,5]. *Av. paragallinarum* is classified into three major serovars (A, B, and C) and exhibits a global distribution, with confirmed reports from numerous countries, including China, the United States, and South Africa [6,7,8,9,10,11,12,13]. Although several virulence factors—such as lipopolysaccharide, hemagglutinin, and fimbriae—have been identified [14,15,16], the comprehensive pathogenic mechanisms of *Av. paragallinarum*, particularly those underlying chronic infection and evasion of host defenses, have remained incompletely elucidated.

Current control strategies primarily involve vaccination and antibiotic treatment. However, vaccine efficacy is frequently constrained by the lack of robust cross-protection among different serovars, and the escalating challenge of antimicrobial resistance further complicates disease management [6,11,13,17,18]. This situation underscores the urgent need to elucidate novel virulence determinants that could serve as targets for the development of more effective vaccines or alternative therapeutic interventions.

Biofilm formation is a critical factor contributing to bacterial persistence, antibiotic recalcitrance, and evasion of host immune clearance in numerous pathogens. Biofilms are structured communities of bacteria encased in a self-produced extracellular polymeric substance (EPS) matrix, a complex hydrogel composed of polysaccharides, proteins, extracellular DNA, and lipids, which can account for over 90% of the biofilm’s dry mass [19,20,21,22,23]. This matrix functions as a formidable physical barrier, conferring mechanical stability and drastically enhancing antibiotic tolerance—often by more than 100-fold—by impeding antimicrobial penetration [24,25,26,27,28,29]. Beyond conferring drug resistance, the biofilm mode of growth serves as a key amplifier of bacterial virulence [30]. It facilitates immune evasion through multiple mechanisms: the sheer size of biofilm aggregates can hinder phagocytosis by immune cells such as neutrophils, and the EPS matrix can mask surface-associated bacterial structures, thereby preventing recognition by the host immune system [31,32,33]. Moreover, the altered metabolic state of bacteria within biofilms promotes the emergence of persister cells, which are central to the establishment of chronic and recurrent infections [34,35,36]. The formation of these complex structures is orchestrated by intricate regulatory networks involving quorum sensing, two-component systems, and other signaling pathways [37,38,39].

Notably, *Av. paragallinarum* has been demonstrated to form biofilms [40,41], suggesting that this phenotype likely constitutes a crucial component of its pathogenicity and may contribute to bacterial persistence as well as the challenges associated with disease control. Despite the recognized importance of biofilm formation, systematic genetic investigations aimed at identifying the specific genes governing this process in *Av. paragallinarum* remain limited. A recent study by Guo et al. [42] constructed a random transposon mutant library comprising approximately 3000 mutants and identified 17 genes associated with impaired biofilm formation, thereby providing the initial genetic blueprint for biofilm development in this pathogen. However, this pioneering work focused exclusively on biofilm-deficient mutants, leaving the regulatory roles of genes whose disruption enhances biofilm formation largely unexplored. A comprehensive understanding of the genetic circuitry governing biofilm formation—including both positive and negative regulators—is therefore essential for fully elucidating the pathogenic mechanisms of *Av. paragallinarum* and for identifying novel targets for therapeutic intervention.

To address this knowledge gap, the present study aimed to systematically identify the genetic determinants involved in biofilm formation in *Av. paragallinarum*, with the objective of not only validating known biofilm-associated genes but also discovering novel regulatory elements. By comprehensively elucidating both positive and negative regulators of biofilm formation and the complex regulatory network they constitute, and by further exploring the intrinsic link between this network and bacterial virulence, we seek to provide new insights into the pathogenic mechanisms of this organism. To achieve this objective, we employed a random transposon mutagenesis approach to construct a high-throughput, whole-genome mutant library [43,44,45,46,47]. Through phenotypic screening, we isolated mutants exhibiting either enhanced or reduced biofilm-forming capacity and systematically evaluated their virulence-associated traits, including adhesion, invasion, and cytotoxicity. This strategy aims to establish a more comprehensive genetic map of biofilm formation in *Av. paragallinarum*, thereby laying a theoretical foundation for a deeper understanding of its pathogenesis and for the development of novel strategies to control infectious coryza.

## 2. Materials and Methods

### 2.1. Strains, Cells, and Culture Conditions

The bacterial strains and plasmids used in this study are listed in Table 1. *Av. paragallinarum* was cultured in tryptic soy broth (TSB) at 37 °C, 180 rpm, or on TSA solid medium containing 1.5% agar at 37 °C under 5% CO_2_ atmosphere. Antibiotics were added as needed at the following concentrations: tetracycline hydrochloride (20 µg/mL) and erythromycin (10 µg/mL). DF-1 cells were cultured in Dulbecco’s Modified Eagle Medium (DMEM, Thermo Fisher Scientific, Waltham, MA, USA) supplemented with 10% fetal bovine serum (FBS) at 37 °C under 5% CO_2_ conditions.

### 2.2. Construction of Av. paragallinarum Transposon Mutant Library

A random transposon mutant library of *Av. paragallinarum* was constructed via triparental conjugation. Escherichia coli strain S17 harboring the transposon plasmid pEVS170 served as the donor strain, *Av. paragallinarum* (strain JZIC-005) as the recipient strain, and *E. coli* harboring plasmid pRK2013 as the helper strain. All three strains were cultured to the logarithmic growth phase. Bacterial pellets were collected, washed with sterile 10 mM MgSO_4_, and mixed at a ratio of donor:recipient:helper of 2:2:1. The mixture was spotted onto TSA plates and incubated upright at 37 °C under 5% CO_2_ for 24 h. Subsequently, the bacterial growth was scraped off, resuspended in 10 mM MgSO_4_, and plated onto TSA screening plates containing tetracycline hydrochloride (20 µg/mL) and erythromycin (10 µg/mL). Plates were incubated at 37 °C under 5% CO_2_ until transconjugants appeared. Potential mutants were confirmed by PCR amplification of the erythromycin resistance gene on the mini-Tn5 transposon and the *Av. paragallinarum*-specific *HagA* gene, while ensuring the absence of the kanamycin resistance gene from the pEVS170 plasmid. Confirmed mutant strains were stored at −80 °C in a solution of 25% glycerol. (Primer sequences are shown in Table 2).

### 2.3. Phenotypic Screening of Biofilm Formation in Mutant Library

Biofilm-forming capacity was assessed using the crystal violet staining method [48]. PCR-confirmed mutant strains and the wild-type strain were cultured in tryptic soy broth (TSB) to an optical density at 600 nm (OD_600_) of 0.6 ± 0.02. Subsequently, 20 μL of each bacterial suspension was inoculated into 180 μL of fresh TSB per well in 96-well polystyrene microtiter plates. Each strain was tested in triplicate. Plates were incubated at 37 °C under 5% CO_2_ and 45% humidity for 24 h. To monitor growth kinetics and account for potential growth-dependent effects on biofilm quantification, OD_600_ was also measured at 14 and 24 h post-inoculation. Following incubation, the culture medium was discarded, and non-adherent bacteria were removed by gently rinsing the wells three times with sterile phosphate-buffered saline (PBS). After air-drying the plates, 200 μL of 0.1% (*w*/*v*) crystal violet solution was added to each well and incubated for 20 min at room temperature. Excess stain was then discarded, and the wells were gently rinsed with PBS. Following complete drying, 200 μL of absolute ethanol was added to each well to solubilize the bound crystal violet, and the plates were incubated for 10 min. The absorbance at 595 nm (OD_595_) was measured using a microplate reader. Mutants exhibiting significantly altered biofilm-forming capacity in the initial screen (*p* < 0.05) were subjected to secondary and tertiary screening rounds to confirm phenotypic stability. Only those mutants displaying significant changes consistently across all three screening rounds (primary, secondary, and tertiary) were selected as candidate strains for subsequent analyses.

Statistical analyses were performed using GraphPad Prism version 9.5 (GraphPad Software, San Diego, CA, USA). Data are presented as mean ± standard deviation. Homogeneity of variances was assessed using the Brown–Forsythe test; however, owing to the limited sample size (*n* = 3 per group), the results of this test should be interpreted with caution. Comparisons among multiple groups were conducted using one-way analysis of variance (ANOVA) followed by Dunnett’s multiple comparisons test to evaluate differences between each experimental group and the control group. A *p*-value < 0.05 was considered statistically significant.

### 2.4. Transposon Insertion Site Identification and Mutant Gene Function Analysis

To minimize the risk of phenotypic alterations arising from secondary mutations or multicopy transposon insertions, all candidate strains were subjected to at least two rounds of single-colony purification prior to genomic DNA extraction and subsequent TAIL-PCR analysis. Moreover, during the analysis of TAIL-PCR products, mutants yielding a single, distinct band were prioritized, whereas those producing excessive bands were excluded to avoid potential confounding effects from multiple insertion sites in downstream experiments.

Thermal asymmetric interlaced PCR (TAIL-PCR) was employed to amplify the genomic regions flanking the transposon insertion sites. Three nested sequence-specific primers with high annealing temperatures (R1, R2, R3; see Table 2) were used in successive PCR rounds in conjunction with degenerate primers (LAD1 and LAC). In the primary round, PCR was performed using primer LAD1 and the specific primer R1, with mutant genomic DNA as the template. The resulting product was diluted and used as the template for the secondary round with primers LAC and R2. The secondary PCR product was further diluted and subjected to a tertiary round of amplification with primers LAC and R3. Products from the secondary and tertiary rounds were resolved by agarose gel electrophoresis, purified, and subjected to Sanger sequencing. The obtained flanking sequences were aligned against the reference genome of *Av. paragallinarum* strain C-AP4 (NCBI accession No. CP113955.1) using BLASTn (https://blast.ncbi.nlm.nih.gov, accessed on 28 February 2026) to identify the disrupted genes. Lists of identified genes were subsequently subjected to functional enrichment analyses, including Gene Ontology (GO), Kyoto Encyclopedia of Genes and Genomes (KEGG), and Clusters of Orthologous Groups (COG) classification. Visualization of the functional annotation results was performed using GraphPad Prism version 9.5.0.

### 2.5. Growth Curve Assay

The growth kinetics of the wild-type strain and selected mutants (Tn-441, Tn-483, Tn-1216, Tn-1504, Tn-1706, Tn-2206, Tn-2428, and Tn-2859) were determined. Overnight cultures were diluted to an OD_600_ of 0.6 and then subcultured at a 1:100 dilution into 50 mL of fresh TSB in a shaking incubator at 37 °C and 180 rpm. The OD_600_ was measured hourly for 14 h.

### 2.6. Adhesion Assay

Bacterial adhesion to host cells was assessed using chicken embryo fibroblast (DF-1) cells. DF-1 cells were seeded into 24-well plates and grown to approximately 70–80% confluency. Bacterial suspensions were added at a multiplicity of infection (MOI) of 100. After a 2-h incubation at 37 °C under 5% CO_2_, the bacterial suspension was removed, and monolayers were gently washed three times with sterile PBS to remove non-adherent bacteria. Cells were lysed with 1 mL of 0.5% Triton X-100 for 10 min. Lysates were serially diluted and plated on TSA supplemented with NAD and chicken serum to enumerate viable colony-forming units (CFU). Data were expressed as mean ± standard deviation. Homogeneity of variances was assessed using the Brown–Forsythe test; however, due to the small sample size (*n* = 2 per group), the results of this test should be interpreted with caution. All data are presented as mean ± SD (*n* = 2). Comparisons with wild-type (WT) were performed using one-way ANOVA followed by Dunnett’s multiple comparison test. *p* < 0.05 was considered statistically significant.

### 2.7. Invasion Assay

Cell invasion capability was evaluated using a gentamicin protection assay. DF-1 cells were infected as described for the adhesion assay. After the 2-h infection and PBS washes, fresh medium containing 25 µg/mL gentamicin was added and incubated for 1 h to kill extracellular bacteria. Cells were then lysed with 0.5% Triton X-100, and intracellular bacteria were quantified by plating as described above. Data were expressed as mean ± standard deviation. Homogeneity of variances was assessed using the Brown–Forsythe test; however, due to the small sample size (*n* = 2 per group), the results of this test should be interpreted with caution. All data are presented as mean ± SD (*n* = 2). Comparisons with wild-type (WT) were performed using one-way ANOVA followed by Dunnett’s multiple comparison test. *p* < 0.05 was considered statistically significant.

### 2.8. Cytotoxicity Assay

Cytotoxicity was assessed by measuring lactate dehydrogenase (LDH) release. DF-1 cells were seeded in a 96-well plate and incubated overnight. Bacterial suspensions were added at an MOI of 1000. To ensure contact, plates were centrifuged at 1000× *g* for 5 min before incubation at 37 °C under 5% CO_2_ for 4 h. Supernatants were collected, and LDH activity was measured using the CytoTox96^®^ Non-Radioactive Cytotoxicity Assay kit (Promega, Madison, WI, USA) according to the manufacturer’s instructions. Each condition was tested in quadruplicate. Cytotoxicity was expressed as a percentage relative to the maximum LDH release control (cells lysed with Triton X-100). Data were expressed as mean ± standard deviation. Homogeneity of variances was assessed using the Brown–Forsythe test; however, due to the small sample size (*n* = 3 per group), the results of this test should be interpreted with caution. All data are presented as mean ± SD (*n* = 3). Comparisons with wild-type (WT) were performed using one-way ANOVA followed by Dunnett’s multiple comparison test. *p* < 0.05 was considered statistically significant.

### 2.9. Statistical Analysis

Data analysis and visualization were performed using GraphPad Prism 9.5.0 software. Statistical significance was determined by one-way analysis of variance (ANOVA) with appropriate post hoc tests, as indicated in the figure legends. Significance levels are denoted as follows: ns, not significant; * *p* < 0.05; ** *p* < 0.01; *** *p* < 0.001; **** *p* < 0.0001.

## 3. Results

### 3.1. Identification of Mutant Strains

PCR results indicate that all mutant strains harbored an erythromycin resistance gene (Erm) of approximately 484 bp and an *Av. paragallinarum*-specific gene (*HagA*) of approximately 510 bp, while no kanamycin resistance gene (Kan) was detected. This indicates erythromycin resistance in mutant strains arises from random integration of the mini-Tn5 transposon element within the pEVS170 vector system, rather than autonomous plasmid transfer. This molecular feature confirms the successful construction of the transposon mutant library. The vast majority of mutant strains screened from dual-resistance medium were transposon insertion-positive random mutants, with only a few exceptions. A total of 3106 random mutant strains were identified and designated Tn1-Tn3106.

### 3.2. Screening of Mutant Strains for Altered Biofilm-Forming Capacity in Av. paragallinarum

**Figure 1:**
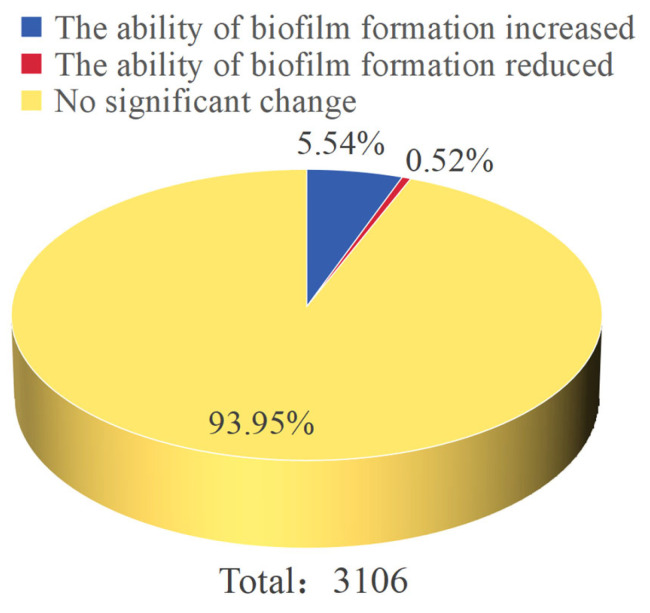
Proportion of mutants with increased or decreased biofilm formation.

**Figure 2:**
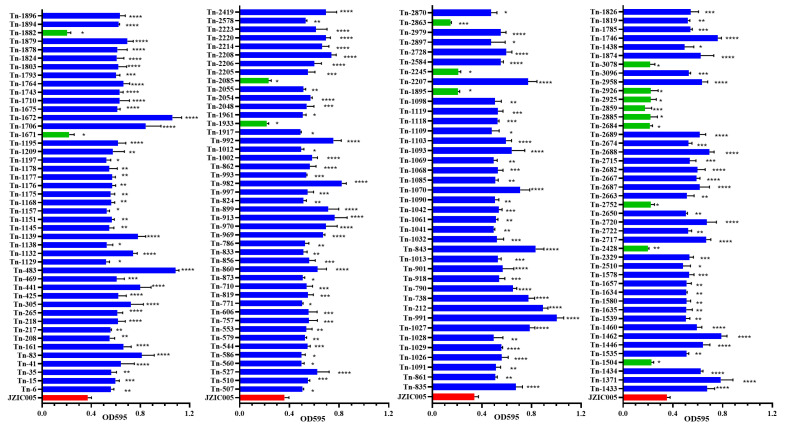
Analysis of biofilm formation among 3106 transposon mutants. Following random transposon mutagenesis, 188 mutant strains exhibited significantly altered biofilm-forming capacity. Biofilm biomass was quantified by measuring the absorbance at 595 nm (OD_595_) of ethanol-solubilized crystal violet. Data are presented as mean ± SD, with each assay representing an independent biofilm formation experiment. Statistical significance compared to the control group was determined by one-way ANOVA with Dunnett’s post hoc test (* *p* < 0.05, ** *p* < 0.01, *** *p* < 0.001, **** *p* < 0.0001 relative to the wild-type control). Quantitative results for selected mutants are shown.

Biofilm formation capacity was evaluated for all 3106 transposon mutant strains via crystal violet staining. The results indicated that 2918 mutants (93.94%) displayed no significant difference in biofilm formation compared to the wild-type strain. In contrast, 172 mutants (5.54%) showed significantly enhanced biofilm formation, while 16 mutants (0.52%) exhibited significantly reduced biofilm formation (Figure 1). Further analysis revealed that the biofilm phenotypes varied in degree among the different mutant strains. Partial statistical results of the OD_595_ measurements are summarized in Figure 2. Mutants with significantly reduced biofilm formation included Tn-1504, Tn-2752, Tn-2684, etc. (*p* < 0.05); Tn-2428 (*p* < 0.01); Tn-2859 (*p* < 0.001). Mutant strains with significantly increased biofilm formation included Tn-1438, Tn-2510 (*p* < 0.05); Tn-1535, Tn-1539, Tn-1635, etc. (*p* < 0.01); Tn-1578, Tn-2329, Tn-2715, etc. (*p* < 0.001); and Tn-1371, Tn-1433, Tn-1462, etc. (*p* < 0.0001).

### 3.3. Identification of Transposon Insertion Sites in Mutant Strains with Altered Biofilm Phenotypes

**Figure 3:**
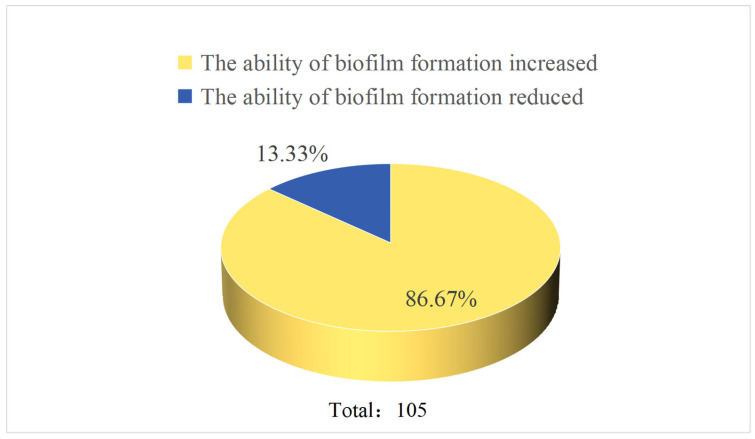
Proportion of genes with increased or decreased biofilm formation.

Given the random nature of transposon insertion, multiple insertions can occur within the same gene. After mapping the insertion sites and removing duplicates, 105 non-redundant genes were identified from 188 mutant strains that showed significant alterations in biofilm formation (Table 3). Of these, 19 genes were annotated as encoding putative or hypothetical proteins. Fourteen unique genes were linked to reduced biofilm formation, whereas 91 were associated with enhanced biofilm formation (Figure 3). The difference between the number of mutant strains (188) and the number of unique genes identified (105) indicates that certain genes were independently mutated in multiple strains.

### 3.4. Bioinformatics Analysis of Proteins Encoded by Inserted Genes

**Figure 4:**
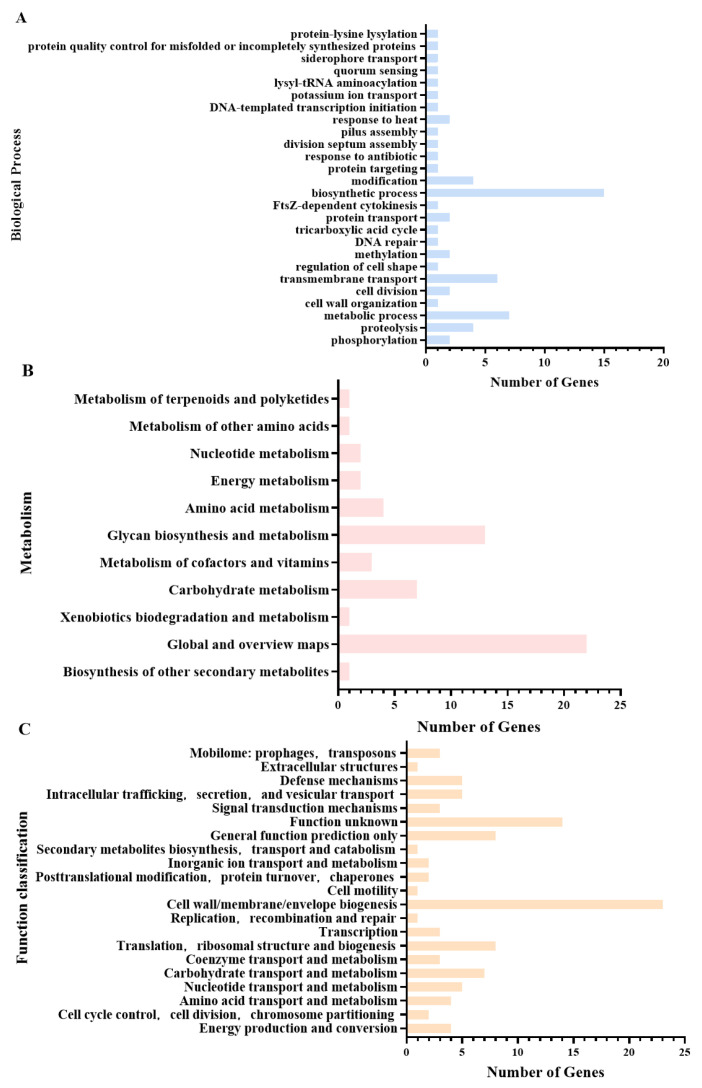
Gene Ontology (GO) enrichment analysis, Kyoto Encyclopedia of Genes and Genomes (KEGG) pathway enrichment analysis, and Clusters of Orthologous Groups (COG) classification were performed on the 105 genes harboring transposon insertions. Bar lengths indicate the number of genes assigned to each respective term, pathway, or category. (**A**) GO enrichment analysis of the 105 genes identified with insertion mutations; (**B**) KEGG pathway enrichment analysis of the 105 genes identified with insertion mutations; (**C**) COG functional classification of the 105 genes identified with insertion mutations.

Gene Ontology (GO) enrichment analysis revealed that the proteins encoded by the genes interrupted by transposon insertions were primarily involved in biosynthesis and metabolism (22 genes), transmembrane transport (6 genes), proteolysis and transport (4 genes), phosphorylation or methylation (4 genes), cell division (2 genes), and ciliary assembly (1 gene) (Figure 4A). Kyoto Encyclopedia of Genes and Genomes (KEGG) pathway analysis further indicated that these genes predominantly participate in sugar biosynthesis and metabolism (13 genes), carbohydrate metabolism (7 genes), and amino acid, cofactor, and vitamin metabolism (7 genes), among other metabolic pathways (Figure 4B). Clusters of Orthologous Groups (COG) functional classification demonstrated that the most abundant categories among these protein-coding genes were cell wall/membrane/envelope biogenesis (23 genes); translation, ribosomal structure, and biogenesis (8 genes); carbohydrate transport and metabolism (7 genes); and intracellular trafficking, secretion, and vesicular transport (5 genes), in addition to other functional classes (Figure 4C). Based on the integration of quantitative biofilm screening data and functional annotation results, a systematic screening of 188 biofilm-associated mutant strains was conducted in this study. The selection criteria were as follows: First, to ensure the reliability of the genetic background, all candidate mutant strains were verified to produce a single band by TAIL-PCR and confirmed to exhibit stable phenotypes through three rounds of independent screening. On this basis, to comprehensively cover biofilm-associated phenotypes and functions, both biofilm-enhanced and biofilm-deficient strains were selected, with priority given to mutants carrying insertions in genes known to be involved in biofilm formation. Additionally, to explore potential novel regulatory factors, two mutants carrying insertions in genes encoding hypothetical proteins were specifically selected. Ultimately, a total of eight representative mutant strains—Tn-441, Tn-483, Tn-1216, Tn-1504, Tn-1706, Tn-2206, Tn-2428, and Tn-2859—were selected for subsequent studies. Detailed information on these mutants is provided in Table 4.

### 3.5. Growth Curves of Biofilm-Related Mutant Strains

**Figure 5:**
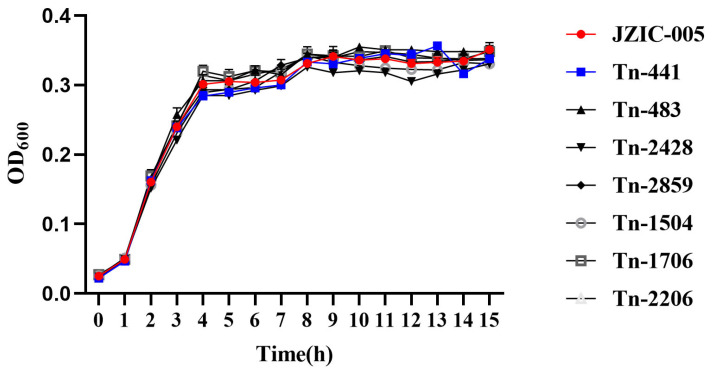
Analysis of bacterial growth curves indicated that the wild-type strain and the mutant strains exhibited highly similar growth kinetics. This figure shows the growth curves of the wild-type strain and its mutants cultured with shaking at 37 °C in TSB medium. The OD_600_ was measured at 1-h intervals. All data are presented as mean ± SD (*n* = 3).

Growth curves were measured for the wild-type strain JZIC-005 and the transposon insertion mutant strains Tn-441, Tn-483, Tn-1216, Tn-1504, Tn-1706, Tn-2206, Tn-2428, and Tn-2859. Comparative analysis revealed that all mutant strains exhibited growth kinetics highly similar to those of the wild-type strain (Figure 5), indicating that the observed alterations in biofilm-forming capacity are not attributable to differences in growth ability.

### 3.6. Adhesion and Invasion Capabilities of Biofilm-Related Mutants on DF-1 Cells

The adhesion and invasion capabilities of the wild-type strain JZIC-005 and the eight biofilm-related mutant strains were evaluated using DF-1 cells.

**Figure 6:**
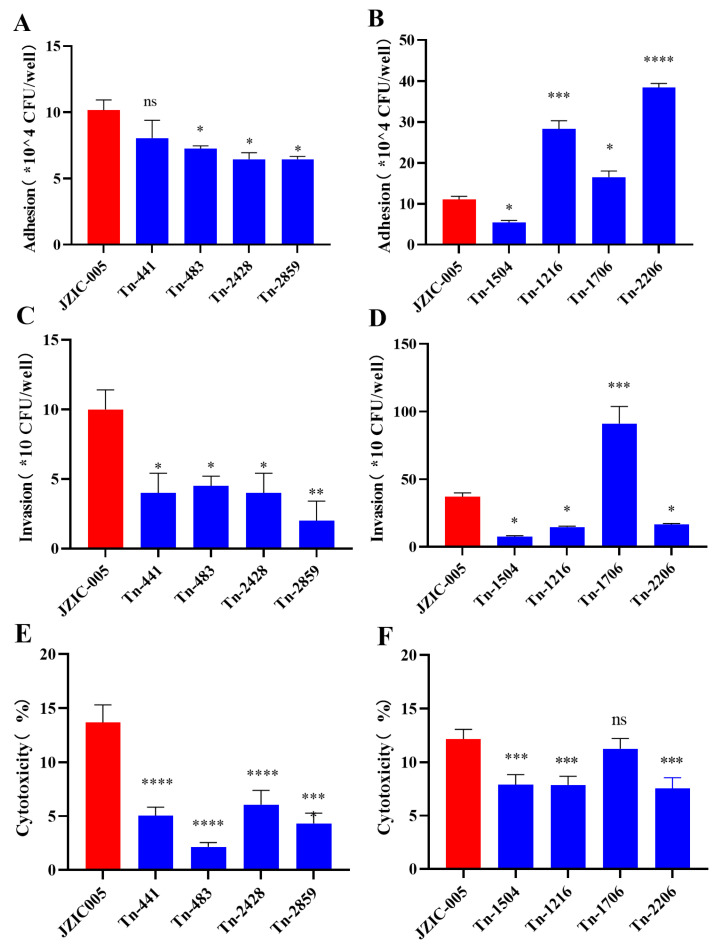
Adhesion, invasion, and cytotoxicity capabilities of mutant strains Tn-483, Tn-2428, and Tn-2859. Compared to the wild-type strain, the adhesive and invasive capabilities, as well as the cytotoxicity, of the three mutant strains were significantly reduced. (**A**,**B**) Bacterial adhesion assay. DF-1 cells were infected with the wild-type and mutant strains at a multiplicity of infection (MOI) of 100 for 2 h, and adherent bacteria were counted. Data are presented as the relative number of adhered bacteria compared to the wild-type. All data are presented as mean ± SD (*n* = 2). (**C**,**D**) Bacterial invasion assay. DF-1 cells were infected with the wild-type and mutant strains at an MOI of 100 for 2 h. Extracellular bacteria were killed by gentamicin treatment to quantify the number of invading bacteria. Data are presented as the relative number of invading bacteria compared to the wild-type. All data are presented as mean ± SD (*n* = 2). (**E**,**F**) Cytotoxicity assay. DF-1 cells were infected with the wild-type and mutant strains at an MOI of 1000 for 4 h. Cytotoxicity was measured using a lactate dehydrogenase (LDH) release assay and expressed as a percentage relative to the maximum LDH release control. All data are presented as mean ± SD (*n* = 3). Statistical significance compared to the wild-type strain was determined by one-way ANOVA followed by Dunnett’s multiple comparison test. ns, not significant; * *p* < 0.05, ** *p* < 0.01, *** *p* < 0.001, and **** *p* < 0.0001.

Adhesion assays revealed significant differences compared to the wild type. The adhesion capacity of mutants Tn-483, Tn-1504, Tn-2428, and Tn-2859 was significantly decreased (*p* < 0.05). Mutant Tn-1504 exhibited the most pronounced reduction, with a 50.68% lower adhesion rate, followed by Tn-2428 and Tn-2859. In contrast, mutants Tn-1216 (*p* < 0.001), Tn-1706 (*p* < 0.05), and Tn-2206 (*p* < 0.0001) showed significantly enhanced adhesion. Notably, the adhesion rate of Tn-2206 increased by 247.51%. No significant difference (*p* > 0.05) was observed for mutant Tn-441 (Figure 6A,B). These findings indicate that transposon insertion into genes *OW731_03300*, *OW731_11000*, and *lsrk* enhances bacterial adhesion to host cells, whereas insertion into *OW731_01055*, *OW731_10585*, *OW731_03180*, and *OW731_09840* impairs this ability.

Invasion assays revealed that several mutants exhibited significant reductions in the number of bacteria invading DF-1 cells compared to the wild type. These included Tn-441, Tn-483, Tn-1504, Tn-1216, Tn-2206, and Tn-2428 (*p* < 0.05), as well as Tn-2859 (*p* < 0.01). The most pronounced defect was observed in mutant Tn-2859, which showed an 80% decrease in invasion, followed by Tn-1504 and Tn-2206. In contrast, mutant Tn-1706 (*p* < 0.001) demonstrated a significantly enhanced capacity to infect DF-1 cells (Figure 6C, D). Collectively, these results suggest that the genes *OW731_05445*, *OW731_01055*, *OW731_03300*, *OW731_10585*, *lsrk*, *OW731_03180*, and *OW731_09840* promote cellular invasion by *Av. paragallinarum*, while the *OW731_11000* gene appears to attenuate this process.

### 3.7. Cytotoxicity Assay of Biofilm Defect Mutant Strains Against DF-1 Cells

The cytotoxicity of the biofilm mutants was evaluated using a lactate dehydrogenase (LDH) release assay. Compared to the wild-type strain, mutants Tn-441, Tn-483, Tn-1216, Tn-1504, Tn-2206, Tn-2428, and Tn-2859 exhibited significantly reduced toxicity toward DF-1 cells. The most pronounced decrease was observed for Tn-2859, which showed 66.38% lower toxicity relative to the wild type, followed by Tn-441 and Tn-2428. In contrast, no significant difference in cytotoxicity was detected for mutant Tn-1706 (*p* > 0.05) (Figure 6E,F). These results indicate that transposon insertion into the genes *OW731_05445*, *OW731_01055*, *OW731_03300*, *OW731_10585*, *lsrk*, *OW731_03180*, and *OW731_09840* reduces the virulence of *Av. paragallinarum*.

## 4. Discussion

In this study, we performed a systematic, genome-wide screen for genes involved in biofilm formation in *Av. paragallinarum*. By integrating transposon mutagenesis with phenotypic screening, we identified a complex genetic network underlying this critical virulence-associated trait and constructed a comprehensive genetic landscape encompassing both positive and negative regulators of biofilm formation. Using mini-Tn5 transposon mutagenesis, we successfully generated a mutant library comprising 3106 individual mutants, which theoretically covers approximately 1.2-fold of the protein-coding sequences in the reference genome CP113955.1 (3106/2592), thereby achieving near-saturation mutagenesis. Initial screening via crystal violet staining identified 188 mutants exhibiting significantly altered biofilm-forming capacity relative to the wild-type strain, including 172 mutants with enhanced and 16 with reduced biofilm formation. Subsequent TAIL-PCR coupled with sequencing analysis successfully identified 105 non-redundant genes disrupted by transposon insertions. The combination of large-scale saturating mutagenesis with systematic phenotype–genotype association analysis strongly supports the comprehensiveness and reliability of the findings presented in this study.

In this study, mutants Tn-483, Tn-2428, and Tn-2859 exhibited reduced biofilm formation, along with diminished adhesion to and invasion of DF-1 cells, as well as attenuated cytotoxicity. Among these, mutant strain Tn-2428, which carries a transposon insertion in the gluconate permease (GntP)-encoding gene (*OW731_03180*), displayed comprehensive attenuation of the aforementioned virulence-related phenotypes, suggesting that disruption of gluconate metabolism resulting from gntP inactivation may underlie this pleiotropic defect. In *E. coli*, GntP is involved in glucuronate transport and is regulated by UxuR; deletion of uxuR impairs biofilm formation [49,50,51]. Moreover, gntP deletion in *E. coli* reduces intestinal colonization capacity [52]. Based on these findings, we hypothesize that disruption of this sugar transporter in *Av. paragallinarum* may alter metabolic flux, which may in turn interfere with biofilm development and virulence expression through effects on energy homeostasis, precursor availability, or signaling molecules. Future metabolomic studies to quantify intracellular gluconate levels could test this hypothesis.

The autoinducer-2 (AI-2) quorum-sensing (QS) system is a crucial intercellular communication mechanism that regulates collective bacterial behaviors, including biofilm formation and virulence expression [38,53,54,55]. Within this pathway, the LsrK kinase plays a pivotal role by phosphorylating internalized AI-2, thereby activating downstream signaling cascades that coordinate pathogenicity traits [56,57,58]. In the present study, the *lsrK* transposon mutant Tn-2206 exhibited a distinctive phenotypic profile, characterized by markedly enhanced biofilm formation and host cell adhesion, yet concurrently reduced cellular invasion and cytotoxicity. This decoupling of phenotypes—in which colonization potential is increased while virulence capacity is diminished—mirrors observations in *Klebsiella pneumoniae*, where deletion of the *lsrCD* gene downregulates *lsrK* expression and yields analogous outcomes [59]. The mechanistic basis for this phenomenon may stem from a dual effect: the primary disruption of the LsrK-mediated QS cascade likely attenuates the expression of acute virulence factors required for invasion and cytotoxicity, while concurrently dysregulating feedback loops within the QS network, leading to compensatory overexpression of genes that promote biofilm maturation and adhesion [57,58]. This dissociation between biofilm-mediated persistence and invasive damage aligns closely with the principles of the “anti-virulence” strategy, which seeks to disarm pathogens rather than eliminate them. Collectively, our findings suggest that LsrK may represent a promising target for the development of quorum-sensing inhibitors against *Av. paragallinarum*, although further functional validation is warranted.

The TonB protein functions as an energy-transducing component in the iron acquisition systems of Gram-negative bacteria and plays an essential role in the transmembrane transport of iron complexes [60,61]. Iron availability serves as an environmental signal that influences biofilm architecture; sufficient iron can activate the expression of biofilm-associated genes, whereas iron limitation may suppress the production of extracellular polymeric substances, resulting in structurally fragile biofilms [62,63]. Consistent with this, TonB-deficient mutants in other pathogens, such as *Riemerella anatipestifer* and *E. coli*, exhibit impaired biofilm formation and reduced colonization capacity [64,65]. In the present study, the Tn-1504 mutant, which carries a transposon insertion in the tonB gene (*OW731_10585*), displayed similar pleiotropic phenotypic alterations, including reduced biofilm formation, diminished adhesion to and invasion of host cells, and attenuated cytotoxicity. These observations suggest that in *Av. paragallinarum*, the TonB system may not only participate in iron acquisition but also influence biofilm matrix synthesis and the expression of additional virulence determinants, potentially through iron-dependent regulatory pathways.

Interestingly, mutations affecting surface structures such as fimbriae can lead to a dissociation between biofilm formation and acute virulence. The TonB system itself has been shown to influence the production of adhesins, including type IV pili, in other organisms [66]. For instance, disruption of genes involved in type IV pilus biogenesis—such as *apfA* in *Actinobacillus pleuropneumoniae* or *pilU* in *Pseudomonas aeruginosa*—has been reported to enhance biofilm formation while simultaneously diminishing invasive virulence [67,68]. In the present study, the Tn-1216 mutant, which carries a transposon insertion in a gene (*OW731_03300*) implicated in fimbrial biogenesis in *Av. paragallinarum*, phenocopied this dissociation: biofilm formation and adhesion were enhanced, whereas invasion and cytotoxicity were reduced. These observations suggest that surface appendages such as fimbriae in *Av. paragallinarum* may possess distinct, separable functions in surface colonization versus host cell invasion and damage delivery.

In addition to metal acquisition and surface structures, another metabolically relevant mutant, Tn-1706, which carries an insertion in a gene encoding a GDP-L-fucosyltransferase (*OW731_11000*), exhibited enhanced biofilm formation, adhesion, and invasion, whereas cytotoxicity remained unchanged. This phenotypic profile contrasts with previous reports in which fucose availability suppressed biofilm formation in *Campylobacter jejuni* and mutation of a homologous fucosyltransferase attenuated virulence in *Pectobacterium carotovorum* [69,70]. Such divergence may suggest a distinct role for fucose metabolism or fucosylated substrates in regulating biofilm formation in *Av. paragallinarum*, or alternatively, may reflect polar effects on the expression of downstream genes within the same operon.

We also characterized mutants carrying disruptions in genes encoding hypothetical proteins. Tn-2859 (*OW731_09840*) exhibited a coordinated reduction in biofilm formation, adhesion, invasion, and cytotoxicity. In contrast, Tn-441 (*OW731_05445*) and Tn-483 (*OW731_01055*) displayed a decoupled phenotype: while virulence-associated traits (invasion and cytotoxicity) were attenuated, biofilm formation was enhanced. These distinct phenotypic profiles suggest that these uncharacterized proteins may be involved in regulating the balance between a biofilm-associated lifestyle and an invasive state in *Av. paragallinarum*; however, their specific roles warrant further investigation.

In summary, this study has preliminarily identified a set of genes potentially involved in the regulation of biofilm formation in *Av. paragallinarum*. We have constructed a preliminary and more comprehensive phenotypic landscape linking specific genetic disruptions—affecting nutrient acquisition (*TonB*), surface structure biogenesis (*fimbriae*), and metabolism-related genes (*GntP*, *fucosyltransferase*)—to alterations in biofilm development and virulence expression. Notably, three mutant strains, Tn-1504, Tn-2428, and Tn-2859, exhibited attenuated virulence in vitro in the absence of apparent growth defects, suggesting their potential as live attenuated vaccine candidates. It should be emphasized that the transposon insertion mutagenesis employed in this study may exert polar effects on downstream genes; therefore, future construction of in-frame deletion and complementation strains is warranted to confirm the specific functions of each gene. Future research directions emerging from this study include evaluating the in vivo protective efficacy of these candidate strains, as well as exploring the therapeutic potential of newly identified targets—such as the LsrK quorum-sensing kinase and the GntP permease—in the development of anti-biofilm or anti-virulence strategies against infectious coryza.

## Figures and Tables

**Table 1:** Bacterial strains and plasmids.

Strain or Plasmid	Relevant Genotype and Property	Source
JZIC-005	Wild-type *Av. Paragallinarum*	this study
*E. coli* S17-1λpir	Random transposon donor cells	this study
*E. coli* DH5αλpir	Random transposon helper cells	this study
pEVS170	Provide the transposon Tn5 to insert into the chromosome of the target strain	Kindly provided by Professor Eric V. Stabb, University of Georgia, USA.
pRK2013	helper plasmid	this study

**Table 2:** Primers used in this study.

Primer	Sequence (5′-3′)	Description	Product Size (bp)
Erm-F	GACGATATTCTCGATTGACC	For amplification Tn5	484
Erm-R	TTAACGACGAAACTGGCTAA	For amplification Tn5	
Kan-F	AAAGGTAGCGTTGCCAATGAT	For amplification Tn5	570
Kan-R	AAGCCGTTTCTGTAATGAAGGAGA	For amplification Tn5	
LAD1	ACGTGTAGGTCGAGCGGCGCVNNNGGAA	For thermal asymmetric PCR to get the Tn5 insert sequence	
LAC	ACGTTTAGCGGCGAG	For thermal asymmetric PCR to get the Tn5 insert sequence	
R1	GCCAGTTTCGTCGTTAAATGCCCTTTACCTGTTCC	For thermal asymmetric PCR to get the Tn5 insert sequence	
R2	GCGTCCGGCGTAGAGGATCTGAAGATCAGC	For thermal asymmetric PCR to get the Tn5 insert sequence	
R3	CAGGAACACTTAACGGCTGACATGGGAATTGGCT	For thermal asymmetric PCR to get the Tn5 insert sequence	
L1	ATTGCTTAAGCTGCCAGCGGAATGCTTTCATCC	For thermal asymmetric PCR to get the Tn5 insert sequence	
L2	GCTCAATCAATCACCGGATCCTCAGTTGTACCCGG	For thermal asymmetric PCR to get the Tn5 insert sequence	
L3	CGATGCCATTGGGATATATCAACGGTGGT	For thermal asymmetric PCR to get the Tn5 insert sequence	

**Table 3:** Characteristics of 188 *Av. paragallinarum* transposon mutants with significantly altered biofilm-forming capacity used in this study.

Mutants	Gene	Description of Gene	Mean ± SD	*p*	Biofilm Formation Ability
Tn-6	*speF*	ornithine decarboxylase SpeF	0.561 ± 0.024	0.0036	increased
Tn-15	*0W731-06770*	MFS transporter	0.598 ± 0.031	0.0002	increased
Tn-35	*OW731-09985*	hypothetical protein	0.562 ± 0.043	0.0032	increased
Tn-41	*OW731-09980*	hypothetical protein	0.642 ± 0.11	<0.0001	increased
Tn-83	*OW731-05730*	NAD(+) kinase	0.811 ± 0.102	<0.0001	increased
Tn-161	*epmA*	elongation factor P—(R)-beta-lysine ligase	0.663 ± 0.062	<0.0001	increased
Tn-208	*0W731-05085*	glycosyltransferase	0.551 ± 0.04	0.0072	increased
Tn-212	*0W731-03815*	hypothetical protein	0.892 ± 0.041	<0.0001	increased
Tn-217	*OW731-05435*	glycosyltransferase	0.557 ± 0.008	0.0047	increased
Tn-218	*lsrC*	autoinducer 2 ABC transporter permease LsrC	0.617 ± 0.06	<0.0001	increased
Tn-265	*lsrC*	autoinducer 2 ABC transporter permease LsrC	0.613 ± 0.044	<0.0001	increased
Tn-305	*envC*	murein hydrolase activator EnvC	0.723 ± 0.102	<0.0001	increased
Tn-425	*wecA*	UDP-N-acetylglucosamine—undecaprenyl-phosphate N-acetylglucosaminephosphotransferase	0.621 ± 0.064	<0.0001	increased
Tn-441	*0W731-05445*	hypothetical protein	0.799 ± 0.088	<0.0001	increased
Tn-469	*envC*	murein hydrolase activator EnvC	0.607 ± 0.062	0.0001	increased
Tn-483	*0W731-01055*	hypothetical protein	1.087 ± 0.028	<0.0001	increased
Tn-507	*0W731-08150*	hypothetical protein	0.506 ± 0.009	0.0138	increased
Tn-510	*0W731-05430*	lytic murein transglycosylase	0.549 ± 0.015	0.0004	increased
Tn-527	*0W731-06830*	autotransporter domain-containing protein	0.624 ± 0.096	<0.0001	increased
Tn-544	*0W731-09980*	hypothetical protein	0.547 ± 0.021	0.0004	increased
Tn-553	*deoC*	deoxyribose-phosphate aldolase	0.537 ± 0.046	0.0011	increased
Tn-560	*0W731-12050*	phage tail protein	0.499 ± 0.02	0.0233	increased
Tn-579	*kdsB*	3-deoxy-manno-octulosonate cytidylyltransferase	0.527 ± 0.012	0.0025	increased
Tn-586	*0W731-12675*	nucleoside triphosphate pyrophosphohydrolase family protein	0.498 ± 0.03	0.0257	increased
Tn-606	*hrpA*	ATP-dependent RNA helicase HrpA	0.554 ± 0.069	0.0002	increased
Tn-710	*0W731-03840*	hypothetical protein	0.54 ± 0.047	0.0008	increased
Tn-738	*tilS*	tRNA lysidine(34) synthetase TilS	0.775 ± 0.051	<0.0001	increased
Tn-757	*0W731-05445*	hypothetical protein	0.557 ± 0.069	0.0002	increased
Tn-771	*0W731-05085*	glycosyltransferase	0.504 ± 0.007	0.0165	increased
Tn-786	*0W731-05445*	hypothetical protein	0.526 ± 0.032	0.0029	increased
Tn-790	*0W731-07795*	O-antigen ligase	0.65 ± 0.031	<0.0001	increased
Tn-819	*0W731-05085*	glycosyltransferase	0.548 ± 0.045	0.0004	increased
Tn-824	*0W731-05085*	glycosyltransferase	0.514 ± 0.023	0.0078	increased
Tn-833	*0W731-00725*	ROK family protein	0.513 ± 0.03	0.0084	increased
Tn-835	*0W731-05085*	glycosyltransferase	0.673 ± 0.054	<0.0001	increased
Tn-843	*0W731-05085*	glycosyltransferase	0.834 ± 0.066	<0.0001	increased
Tn-856	*0W731-03300*	fimbrial biogenesis outer membrane usher protein	0.561 ± 0.048	0.0001	increased
Tn-860	*rfaD*	ADP-glyceromanno-heptose 6-epimerase	0.627 ± 0.072	<0.0001	increased
Tn-861	*0W731-05085*	glycosyltransferase	0.508 ± 0.016	0.0028	increased
Tn-862	*0W731-00230*	hypothetical protein	0.565 ± 0.05	<0.0001	increased
Tn-873	*ftsI*	peptidoglycan glycosyltransferase FtsI	0.509 ± 0.016	0.0115	increased
Tn-899	*0W731-04595*	hypothetical protein	0.715 ± 0.083	<0.0001	increased
Tn-901	*nrfC*	cytochrome c nitrite reductase Fe-S protein	0.567 ± 0.09	<0.0001	increased
Tn-913	*0W731-03315*	VOC family protein	0.765 ± 0.1	<0.0001	increased
Tn-918	*0W731-05085*	glycosyltransferase	0.539 ± 0.046	0.0002	increased
Tn-969	*0W731-08855*	hemagglutinin repeat-containing protein	0.671 ± 0.016	<0.0001	increased
Tn-970	*0W731-03580*	pyrroline-5-carboxylate reductase	0.695 ± 0.091	<0.0001	increased
Tn-982	*0W731-09615*	monovalent cation:proton antiporter-2 (CPA2) family protein	0.823 ± 0.036	<0.0001	increased
Tn-991	*0W731-09975*	restriction endonuclease subunit S	1.004 ± 0.055	<0.0001	increased
Tn-992	*0W731-09975*	restriction endonuclease subunit S	0.755 ± 0.062	<0.0001	increased
Tn-993	*0W731-09975*	restriction endonuclease subunit S	0.538 ± 0.005	0.001	increased
Tn-997	*0W731-09975*	restriction endonuclease subunit S	0.547 ± 0.048	0.0004	increased
Tn-1002	*0W731-06830*	autotransporter domain-containing protein	0.584 ± 0.042	<0.0001	increased
Tn-1012	*0W731-09975*	restriction endonuclease subunit S	0.495 ± 0.023	0.0318	increased
Tn-1013	*0W731-09975*	restriction endonuclease subunit S	0.529 ± 0.026	0.0005	increased
Tn-1026	*0W731-09975*	restriction endonuclease subunit S	0.56 ± 0.052	<0.0001	increased
Tn-1027	*0W731-09975*	restriction endonuclease subunit S	0.785 ± 0.041	<0.0001	increased
Tn-1028	*0W731-09975*	restriction endonuclease subunit S	0.495 ± 0.074	0.0076	increased
Tn-1029	*0W731-09975*	restriction endonuclease subunit S	0.557 ± 0.011	<0.0001	increased
Tn-1032	*0W731-09975*	restriction endonuclease subunit S	0.521 ± 0.055	0.0009	increased
Tn-1041	*0W731-09975*	restriction endonuclease subunit S	0.494 ± 0.011	0.0084	increased
Tn-1042	*0W731-09975*	restriction endonuclease subunit S	0.536 ± 0.027	0.0003	increased
Tn-1061	*0W731-09975*	restriction endonuclease subunit S	0.512 ± 0.017	0.0019	increased
Tn-1068	*0W731-09975*	restriction endonuclease subunit S	0.529 ± 0.041	0.0005	increased
Tn-1069	*0W731-09975*	restriction endonuclease subunit S	0.494 ± 0.029	0.0084	increased
Tn-1070	*0W731-09975*	restriction endonuclease subunit S	0.709 ± 0.077	<0.0001	increased
Tn-1085	*0W731-05445*	hypothetical protein	0.509 ± 0.023	0.0025	increased
Tn-1090	*0W731-09975*	restriction endonuclease subunit S	0.506 ± 0.031	0.0033	increased
Tn-1091	*0W731-09975*	restriction endonuclease subunit S	0.513 ± 0.038	0.0018	increased
Tn-1093	*0W731-09975*	restriction endonuclease subunit S	0.641 ± 0.104	<0.0001	increased
Tn-1098	*0W731-12630*	SPFH domain-containing protein	0.504 ± 0.052	0.0037	increased
Tn-1103	*0W731-09975*	restriction endonuclease subunit S	0.595 ± 0.045	<0.0001	increased
Tn-1109	*0W731-09975*	restriction endonuclease subunit S	0.479 ± 0.06	0.0246	increased
Tn-1118	*0W731-09975*	restriction endonuclease subunit S	0.526 ± 0.012	0.0006	increased
Tn-1119	*0W731-09975*	restriction endonuclease subunit S	0.53 ± 0.041	0.0005	increased
Tn-1129	*rpoH*	RNA polymerase sigma factor RpoH	0.522 ± 0.027	0.0454	increased
Tn-1132	*0W731-03755*	hypothetical protein	0.74 ± 0.035	<0.0001	increased
Tn-1138	*rpoH*	RNA polymerase sigma factor RpoH	0.527 ± 0.049	0.0338	increased
Tn-1139	*rpoH*	RNA polymerase sigma factor RpoH	0.78 ± 0.064	<0.0001	increased
Tn-1145	*0W731-01300*	hypothetical protein	0.546 ± 0.038	0.0097	increased
Tn-1151	*ThiI*	tRNA 4-thiouridine(8) synthase ThiI	0.567 ± 0.021	0.0023	increased
Tn-1157	*0W731-12440*	hypothetical protein	0.527 ± 0.022	0.0338	increased
Tn-1168	*0W731-05410*	WYL domain-containing protein	0.563 ± 0.028	0.003	increased
Tn-1175	*0W731-09695*	cell division ATP-binding protein FtsE	0.558 ± 0.034	0.0044	increased
Tn-1176	*0W731-09285*	hypothetical protein	0.569 ± 0.029	0.0019	increased
Tn-1177	*0W731-08765*	N-6 DNA methylase	0.571 ± 0.026	0.0016	increased
Tn-1178	*0W731-05440*	glycosyltransferase family 2 protein	0.547 ± 0.066	0.0093	increased
Tn-1194	*0W731-11350*	hypothetical protein	0.636 ± 0.055	<0.0001	increased
Tn-1195	*0W731-06610*	ankyrin repeat domain-containing protein	0.617 ± 0.065	<0.0001	increased
Tn-1197	*0W731-09860*	penicillin-binding protein 1A	0.523 ± 0.035	0.0412	increased
Tn-1202	*0W731-05295*	acetyltransferase	0.623 ± 0.067	<0.0001	increased
Tn-1209	*0W731-05500*	transposase	0.576 ± 0.094	0.0011	increased
Tn-1216	*0W731-03300*	fimbrial biogenesis outer membrane usher protein	0.595 ± 0.079	<0.0001	increased
Tn-1239	*neuB*	N-acetylneuraminate synthase	0.485 ± 0.018	0.0002	increased
Tn-1272	*0W731-03015*	VWA domain-containing protein	0.45 ± 0.042	0.0027	increased
Tn-1273	*0W731-03015*	VWA domain-containing protein	0.489 ± 0.025	0.0001	increased
Tn-1274	*0W731-03015*	VWA domain-containing protein	0.526 ± 0.038	<0.0001	increased
Tn-1275	*0W731-03015*	VWA domain-containing protein	0.43 ± 0.05	0.012	increased
Tn-1286	*0W731-11000*	GDP-L-fucose synthase	0.431 ± 0.04	0.0107	increased
Tn-1297	*0W731-02485*	L,D-transpeptidase family protein	0.456 ± 0.028	0.0017	increased
Tn-1361	*0W731-04170*	DUF2726 domain-containing protein	0.46 ± 0.041	0.0012	increased
Tn-1362	*0W731-05105*	glycosyltransferase family 2 protein	0.459 ± 0.045	0.0013	increased
Tn-1369	*Gmd*	GDP-mannose 4,6-dehydratase	0.491 ± 0.036	0.0001	increased
Tn-1371	*0W731-04170*	DUF2726 domain-containing protein	0.786 ± 0.097	<0.0001	increased
Tn-1373	*0W731-03435*	AEC family transporter	0.458 ± 0.046	0.0014	increased
Tn-1433	*Lon*	endopeptidase La	0.677 ± 0.059	<0.0001	increased
Tn-1434	*0W731-06350*	NAD(P)/FAD-dependent oxidoreductase	0.624 ± 0.019	<0.0001	increased
Tn-1438	*Lon*	endopeptidase La	0.494 ± 0.075	0.0129	increased
Tn-1443	*0W731-12675*	nucleoside triphosphate pyrophosphohydrolase family protein	0.586 ± 0.016	<0.0001	increased
Tn-1446	*neuB*	N-acetylneuraminate synthase	0.643 ± 0.055	<0.0001	increased
Tn-1460	*0W731-01300*	hypothetical protein	0.593 ± 0.04	<0.0001	increased
Tn-1462	*Lon*	endopeptidase La	0.791 ± 0.045	<0.0001	increased
Tn-1504	*0W731-10585*	energy transducer TonB	0.228 ± 0.018	0.0467	reduced
Tn-1535	*0W731-02980*	DNA primase	0.509 ± 0.019	0.0036	increased
Tn-1539	*0W731-02980*	DNA primase	0.505 ± 0.032	0.0053	increased
Tn-1578	*0W731-05100*	nucleotide sugar dehydrogenase	0.531 ± 0.039	0.0005	increased
Tn-1580	*0W731-05080*	hypothetical protein	0.51 ± 0.033	0.0034	increased
Tn-1634	*0W731-11000*	GDP-L-fucose synthase	0.509 ± 0.007	0.0038	increased
Tn-1635	*ThiI*	tRNA 4-thiouridine(8) synthase ThiI	0.508 ± 0.048	0.0039	increased
Tn-1657	*0W731-11000*	GDP-L-fucose synthase	0.511 ± 0.037	0.003	increased
Tn-1671	*0W731-05515*	FtsX-like permease family protein	0.22 ± 0.041	0.0421	reduced
Tn-1672	*0W731-08095*	PDDEXK nuclease domain-containing protein	1.062 ± 0.074	<0.0001	increased
Tn-1675	*0W731-08095*	PDDEXK nuclease domain-containing protein	0.612 ± 0.023	<0.0001	increased
Tn-1706	*0W731-11000*	GDP-L-fucose synthase	0.844 ± 0.124	<0.0001	increased
Tn-1710	*0W731-11000*	GDP-L-fucose synthase	0.632 ± 0.079	<0.0001	increased
Tn-1743	*0W731-11000*	GDP-L-fucose synthase	0.632 ± 0.027	<0.0001	increased
Tn-1746	*0W731-11000*	GDP-L-fucose synthase	0.762 ± 0.029	<0.0001	increased
Tn-1764	*0W731-10990*	sugar phosphate nucleotidyltransferase	0.662 ± 0.051	<0.0001	increased
Tn-1785	*0W731-03015*	VWA domain-containing protein	0.541 ± 0.016	0.0002	increased
Tn-1793	*0W731-12070*	phage tail tape measure protein	0.603 ± 0.031	0.0001	increased
Tn-1803	*0W731-01140*	TIGR01777 family oxidoreductase	0.621 ± 0.067	<0.0001	increased
Tn-1819	*0W731-00230*	hypothetical protein	0.521 ± 0.016	0.0012	increased
Tn-1824	*0W731-00235*	SEL1-like repeat protein	0.608 ± 0.058	<0.0001	increased
Tn-1826	*0W731-01140*	TIGR01777 family oxidoreductase	0.543 ± 0.063	0.0002	increased
Tn-1874	*0W731-03015*	VWA domain-containing protein	0.624 ± 0.105	<0.0001	increased
Tn-1878	*0W731-03015*	VWA domain-containing protein	0.613 ± 0.08	<0.0001	increased
Tn-1879	*0W731-03015*	VWA domain-containing protein	0.695 ± 0.047	<0.0001	increased
Tn-1882	*0W731-03830*	VENN motif pre-toxin domain-containing protein	0.204 ± 0.03	0.0157	reduced
Tn-1894	*0W731-03015*	VWA domain-containing protein	0.623 ± 0.008	<0.0001	increased
Tn-1895	*secD*	protein translocase subunit SecD	0.207 ± 0.014	0.0387	reduced
Tn-1896	*0W731-03015*	VWA domain-containing protein	0.633 ± 0.044	<0.0001	increased
Tn-1917	*OW731-05435*	glycosyltransferase	0.49 ± 0.007	0.046	increased
Tn-1933	*0W731-04805*	sodium:alanine symporter family protein	0.219 ± 0.015	0.0157	reduced
Tn-1961	*0W731-01140*	TIGR01777 family oxidoreductase	0.507 ± 0.028	0.0131	increased
Tn-2048	*0W731-01140*	TIGR01777 family oxidoreductase	0.541 ± 0.057	0.0008	increased
Tn-2054	*0W731-01140*	TIGR01777 family oxidoreductase	0.571 ± 0.013	<0.0001	increased
Tn-2055	*0W731-01140*	TIGR01777 family oxidoreductase	0.514 ± 0.018	0.0078	increased
Tn-2085	*0W731-10802*	phage tail protein	0.233 ± 0.021	0.045	reduced
Tn-2205	*0W731-03015*	VWA domain-containing protein	0.55 ± 0.056	0.0003	increased
Tn-2206	*LsrK*	autoinducer-2 kinase	0.601 ± 0.057	<0.0001	increased
Tn-2207	*Mdh*	malate dehydrogenase	0.772 ± 0.072	<0.0001	increased
Tn-2208	*PurF*	amidophosphoribosyltransferase	0.738 ± 0.039	<0.0001	increased
Tn-2214	*0W731-00215*	UbiX family flavin prenyltransferase	0.664 ± 0.055	<0.0001	increased
Tn-2220	*0W731-07185*	HlyD family efflux transporter periplasmic adaptor subunit	0.694 ± 0.036	<0.0001	increased
Tn-2223	*0W731-03815*	hypothetical protein	0.615 ± 0.088	<0.0001	increased
Tn-2245	*XthA*	exodeoxyribonuclease III	0.21 ± 0.02	0.0472	reduced
Tn-2329	*0W731-03750*	hypothetical protein	0.534 ± 0.032	0.0004	increased
Tn-2419	*xylF*	D-xylose ABC transporter substrate-binding protein	0.695 ± 0.086	<0.0001	increased
Tn-2428	*0W731-03180*	GntP family permease	0.201 ± 0.007	0.0059	reduced
Tn-2510	*0W731-06770*	MFS transporter	0.481 ± 0.06	0.0336	increased
Tn-2578	*0W731-09970*	class I SAM-dependent DNA methyltransferase	0.532 ± 0.01	0.0017	increased
Tn-2584	*0W731-08855*	hemagglutinin repeat-containing protein	0.552 ± 0.021	<0.0001	increased
Tn-2650	*TsaA*	(N6-threonylcarbamoyladenosine(37)-N6)-methyltransferase TrmO	0.506 ± 0.012	0.005	increased
Tn-2663	*0W731-11800*	hypothetical protein	0.514 ± 0.057	0.0024	increased
Tn-2667	*0W731-00760*	NCS2 family permease	0.591 ± 0.028	<0.0001	increased
Tn-2674	*0W731-05375*	50S ribosome-binding GTPase	0.525 ± 0.029	0.0009	increased
Tn-2682	*0W731-02950*	hypothetical protein	0.597 ± 0.063	<0.0001	increased
Tn-2684	*0W731-03605*	DUF262 domain-containing HNH endonuclease family protein	0.216 ± 0.021	0.0195	reduced
Tn-2687	*0W731-08820*	SNF2-related protein	0.615 ± 0.08	<0.0001	increased
Tn-2688	*0W731-02855*	Bax inhibitor-1/YccA family protein	0.695 ± 0.037	<0.0001	increased
Tn-2689	*0W731-02400*	NAD(P)H nitroreductase	0.616 ± 0.048	<0.0001	increased
Tn-2715	*Ppx*	exopolyphosphatase	0.536 ± 0.05	0.0003	increased
Tn-2717	*0W731-04670*	insulinase family protein	0.669 ± 0.036	<0.0001	increased
Tn-2720	*XylG*	D-xylose ABC transporter ATP-binding protein	0.673 ± 0.078	<0.0001	increased
Tn-2722	*TrpA*	tryptophan synthase subunit alpha	0.523 ± 0.028	0.001	increased
Tn-2728	*0W731-05445*	hypothetical protein	0.597 ± 0.045	<0.0001	increased
Tn-2752	*0W731-03620*	protein phosphatase 2C domain-containing protein	0.223 ± 0.029	0.0328	reduced
Tn-2859	*0W731-09840*	hypothetical protein	0.175 ± 0.054	0.0006	reduced
Tn-2863	*0W731-03605*	DUF262 domain-containing HNH endonuclease family protein	0.148 ± 0.006	0.0004	reduced
Tn-2870	*0W731-08150*	hypothetical protein	0.475 ± 0.047	0.0339	reduced
Tn-2885	*0W731-03610*	DUF262 domain-containing protein	0.22 ± 0.056	0.0263	increased
Tn-2897	*srC*	autoinducer 2 ABC transporter permease LsrC	0.473 ± 0.115	0.0371	increased
Tn-2925	*0W731-06720*	chondroitinase family polysaccharide lyase	0.222 ± 0.045	0.0298	reduced
Tn-2926	*0W731-06720*	chondroitinase family polysaccharide lyase	0.222 ± 0.06	0.0298	reduced
Tn-2958	*0W731-03815*	hypothetical protein	0.636 ± 0.045	<0.0001	increased
Tn-2979	*0W731-02980*	DNA primase	0.553 ± 0.042	<0.0001	increased
Tn-3078	*0W731-03785*	VENN motif pre-toxin domain-containing protein	0.217 ± 0.039	0.0205	reduced
Tn-3096	*0W731-03750*	hypothetical protein	0.528 ± 0.015	0.0007	increased

**Table 4:** Characteristics of eight key biofilm mutants.

Mutants	Gene	Description of Gene	Mean ± SD	*p*	Biofilm Formation Ability
Tn-441	*0W731-05445*	hypothetical protein	0.799 ± 0.088	<0.0001	increased
Tn-483	*0W731-01055*	hypothetical protein	1.087 ± 0.028	<0.0001	increased
Tn-1216	*0W731-03300*	fimbrial biogenesis outer membrane usher protein	0.595 ± 0.079	<0.0001	increased
Tn-1504	*0W731-10585*	energy transducer TonB	0.228 ± 0.018	0.0467	reduced
Tn-1706	*0W731-11000*	GDP-L-fucose synthase	0.844 ± 0.124	<0.0001	increased
Tn-2206	*LsrK*	autoinducer-2 kinase	0.601 ± 0.057	<0.0001	increased
Tn-2428	*0W731-03180*	GntP family permease	0.201 ± 0.007	0.0059	reduced
Tn-2859	*0W731-09840*	hypothetical protein	0.175 ± 0.054	0.0006	reduced

## Data Availability

The data presented in this study are available in a public repository (DOI: 10.57760/sciencedb.30806).
